# A 29-Year-Old Patient With Patau Syndrome: A Case Report on Medical Management

**DOI:** 10.7759/cureus.51471

**Published:** 2024-01-01

**Authors:** Shirley M Gandhi, Pruthvi Patel, Taylor Carter, Larry Stutts

**Affiliations:** 1 Internal Medicine - Pediatrics, Alabama College of Osteopathic Medicine, Dothan, USA; 2 Internal Medicine, Alabama College of Osteopathic Medicine, Dothan, USA; 3 Obstetrics and Gynecology, Edward Via College of Osteopathic Medicine, Auburn, USA; 4 Obstetrics and Gynecology, Helen Keller Hospital, Sheffield, USA

**Keywords:** trisomy 13, mosaic trisomy 13, rare genetic diseases, optimal medical management, anesthesia management, laparoscopic assisted vaginal hysterectomy, patau syndrome

## Abstract

Patau syndrome (trisomy 13) is a chromosomal abnormality with multiple malformations due to an additional copy of chromosome 13. This genetic condition has a systemic impact on the development of the human body, which can result in, but is not limited to, microphthalmia, microcephaly, low-set ears, cleft palate, cardiac abnormalities, and abdominal wall defects. It is associated with severe physical and intellectual disabilities and a limited lifespan.

Here, we present a 29-year-old female with a high suspicion of the mosaic form of Patau syndrome. She decided to opt for an elective robotic-assisted vaginal hysterectomy (RAVH) due to worsening menorrhagia and recurrent miscarriages. In addition, the importance of medical interventions from surgery to anesthesia is discussed, with their role in improving the quality of life of the patient.

## Introduction

Trisomy 13 was first described as a chromosomal aneuploidy by Dr. Patau in 1960 [1]. It is characterized by the presence of three copies of chromosome 13, producing an amalgamation of symptoms such as microphthalmia, microcephaly, low-set ears, cleft lip, cleft palate, holoprosencephaly, polydactyly, cutis aplasia, congenital heart disease, polycystic kidney disease, and omphalocele [1-4]. The most common cause of Patau syndrome is the nondisjunction of chromosome 13 during meiosis, leading to midline defects that are often incompatible with life [2]. Patau syndrome can also be due to an unbalanced Robertsonian translocation t(13;14) [2]. The least common cause of Patau syndrome is mosaicism, where some cells in the body have three copies of chromosome 13, while others do not [4]. Only 5% of cases have the mosaic form of trisomy 13, which tends to have a better prognosis with a limited impact on intellectual disabilities [4].

Trisomy 13 occurs in one in 10,000-20,000 live births, with the majority of patients dying in utero [1]. Median survival for individuals with Patau syndrome who survive childbirth is seven to 10 days and 90% die before the age of one [1]. According to a recent study in Japan, intensive management such as resuscitation and surgical intervention to these patients can extend their life expectancy to 733 days [3].

## Case presentation

**Figure 1:**
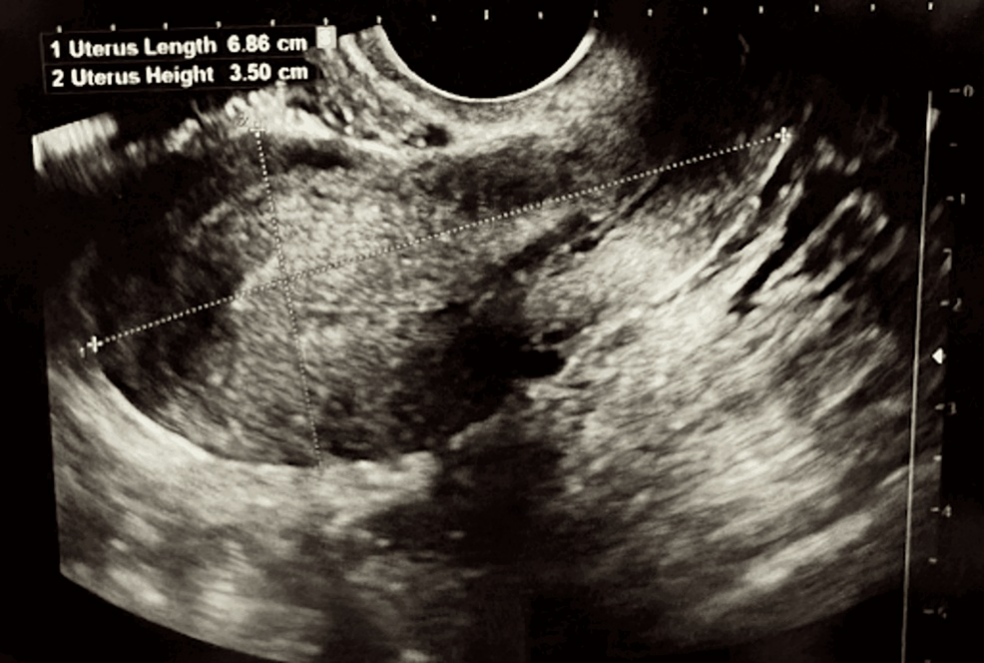
Ultrasound of the patient’s uterus measuring 6.86 x 4.19 x 3.50 cm.

Here, we present a 29-year-old female, with a medical history significant for the mosaic form of Patau syndrome, which was diagnosed a few months after birth. She explained that according to the genetics clinic, she is the longest trisomy 13 survivor in their records. The patient presented to the hospital for a scheduled hysterectomy due to worsening menorrhagia and recurrent miscarriages. She had one spontaneous abortion at 19 weeks and the others between six and 11 weeks. She has had three dilation and curettage (D&C) procedures and is at significant risk of having genetically abnormal pregnancies and increased risks of problems with a pregnancy. She has no desire for future fertility and has failed conservative therapy with Nexplanon, oral contraceptive pills, and intrauterine devices. During the pelvic examination, the uterus was anteflexed, no adnexal masses were palpable, and minimal uterine descensus was present. During the operation, there was normal upper abdominal anatomy but a small uterus (Figure 1). She also presented with elongated ovaries bilaterally but no specific gross lesions were found upon inspection. A robotic-assisted vaginal hysterectomy (RAVH) was conducted and ovaries were left in place since there was no abnormality. The cervix displayed mild to moderate dysplasia and cervical intraepithelial dysplasia (CIN) stages 1-2, which was widely excised. The patient tolerated the procedure well and was taken to the recovery room in stable condition.

With the patient’s history of Patau syndrome, special consideration was taken regarding anesthesiology and securing the airway. A glidescope was utilized to minimize damage to surrounding anatomy and for direct visualization of the airway. A size 7.0 endotracheal tube with stylet was introduced into the trachea with appropriate placement on the first attempt. The appropriate position was confirmed by direct visualization of vocal cord passage, fogging of the tube, and visualized symmetric chest rising. The patient tolerated the procedure well and proceeded under normal conditions for the rest of the surgery. The patient was transferred to the post-anesthesia care unit (PACU) and recovered well without respiratory complications.

## Discussion

Individuals with trisomy 13 are born with congenital anomalies that drastically decrease their survival rate. The correctional surgeries they undergo can be physically and mentally taxing; therefore, the advantages and disadvantages must be thoroughly assessed to optimize their quality of life. In this ethical dilemma, it is critical to acknowledge that a diagnosis of Patau syndrome alone is not enough to make the patient ineligible for correctional procedures [5]. In this case, the patient was constantly dealing with worsening menorrhagia and recurrent miscarriages, which were poorly controlled with medical management, leaving hysterectomy as the only definitive treatment. At the age of five, she had a palatoplasty followed by extensive speech therapy, which improved her ability to communicate with others [5]. At the age of 13, spinal decompression surgery was conducted to remove the T1 vertebrae to improve the patient’s intense neuropathy [6]. In all these cases, various conservative medical interventions were discussed with the patient, but to improve her day-to-day life, aggressive care was deemed necessary.

In regards to anesthesiology, a glidescope should be utilized in all intubation procedures, as patients with Patau syndrome have an increased risk of difficult airways, secondary to possible oral-facial-maxillary surgeries and increased incidence of scoliosis [7]. The patient’s history of severe scoliosis, severe cervical stenosis, T1 laminectomy, and an increased risk of potential anterior airway, warranted the use of a glidescope for intubation. Additionally, the patient had a history of palatoplasty, which increases the patient's risk of adverse airway events [8]. Therefore, utilizing the glidescope was an essential factor in protecting the patient from damage to her airway while also reducing movement to her cervical and thoracic vertebrae. Patients with Patau syndrome may have a smaller or more anterior airway, and therefore the smallest adult intubation tube should be utilized, if possible [7]. Providers may consider the utilization of a pediatric tube if deemed medically appropriate or necessary [7]. Special care should be taken during the removal of the endotracheal tube to minimize damage to any surrounding anatomy, especially in patients with any history of oral-facial surgery. After the procedure, the patient should be closely monitored post-anesthesia for any respiratory complications and quick intervention should be taken if encountered [7].

## Conclusions

Trisomy 13 is a chromosomal aneuploidy that has a low survival rate. The mosaic form of the disease is thought to have a better prognosis, which can be further improved with aggressive medical interventions in all dimensions from anesthesia to surgery. It is critical to balance improving the patient’s day-to-day life and the complications associated with the surgery. Despite such daunting odds, interventional medicine has managed to help improve and extend the patient's quality of life.
